# Association between TBPH exposure, increased MMP9 expression, and atherosclerosis: evidence from integrated network analysis and *in vivo* experiments

**DOI:** 10.3389/fphar.2026.1884077

**Published:** 2026-08-28

**Authors:** Huichao Pan, Dong Wang, Shengguang Chen, Jun Zhou, Lei Song, Min Zhang

**Affiliations:** 1 Division of Cardiology, Institute of Cardiovascular Diseases, Tongren Hospital, Shanghai Jiao Tong University School of Medicine, Shanghai, China; 2 Department of Endocrinology and Metabolism, Gongli Hospital of Shanghai Pudong New Area, Shanghai, China

**Keywords:** atherosclerosis, bis(2-ethylhexyl)-2,3,4,5-tetrabromophthalate, machine learning, MMP9, molecular docking, molecular dynamics simulation, network toxicology

## Abstract

**Introduction:**

Bis(2-ethylhexyl)-2,3,4,5-tetrabromophthalate (TBPH) is used as a nonreactive brominated flame retardant used in industrial products, particularly in flexible polyvinyl chloride and rigid and flexible polyurethane foams. Although TBPH exposure poses potential health risks, its association with atherosclerosis remains poorly understood.

**Methods:**

This study integrated network toxicology, machine learning, molecular docking, and molecular dynamics (MD) simulations to elucidate the influence of TBPH on high-fat diet–induced atherosclerosis.

**Results:**

TBPH exhibited significant predicted toxicity across multiple organs. In total, 167 potential TBPH targets and 690 atherosclerosis-associated targets were identified, with 108 overlapping targets obtained through intersectional analysis. A PPI network was constructed based on these 108 overlapping genes, and the top 20 hub genes were identified. In a parallel independent analysis using a public transcriptomic dataset, 111 differentially expressed genes (DEGs) were identified by comparing atherosclerotic and normal tissues. Subsequent intersection analysis of the 108 overlapping targets and 111 DEGs identified candidate genes that were both TBPH-related and differentially expressed in atherosclerosis. Public transcriptomic data showed higher matrix metalloproteinase 9 (MMP9) level in atherosclerotic tissues. The corresponding receiver operating characteristic (ROC) curve yielded an AUC of 0.806 (95% confidence interval [CI]: 0.698–0.914). Molecular docking and MD simulations indicated favorable predicted binding modes and computational stability between TBPH and MMP9. In vivo experiments further demonstrated an association between TBPH exposure, increased MMP9 expression, and high–fat diet–induced atherosclerotic lesions.

**Discussion:**

These findings suggest that TBPH may be associated with high-fat diet–induced atherosclerosis progression through the dysregulation of key mediators, providing insight into environmental pollutant–driven disease development.

## 1 Introduction

Bis(2-ethylhexyl)-2,3,4,5-tetrabromophthalate (TBPH), a relatively new brominated flame retardant, is ubiquitous in the environment and prone to bioaccumulation. TBPH is widely detected in indoor dust and wildlife; concentrations in urban indoor dust in Beijing range from 260 to 13,000 ng/g (Xiong et al., 2019). Human exposure to TBPH occurs via inhalation, oral ingestion, and dermal absorption (Ragnarsdottir et al., 2022; Li Y. et al., 2025; Persson et al., 2025). TBPH accumulates in human tissues, including hair, fingernails, and breast milk (Zhou et al., 2014; Liu et al., 2016). Emerging evidence indicates that TBPH induces hepatotoxicity (Zhang et al., 2022), reproductive toxicity (Fu et al., 2023), and neurotoxicity (Xiong et al., 2024) in zebrafish and rodent models, making TBPH a globally prevalent environmental contaminant with incompletely characterized cardiovascular hazards.

Atherosclerosis is a chronic inflammatory disease of the arteries (Miano et al., 2021; Döring et al., 2024) that is shaped by crosstalk between genetic and environmental factors (Hahad et al., 2025; Seneviratne et al., 2025). Exposure to toxicants can remodel vascular epigenetics and gene expression, thereby weakening arterial walls and exacerbating vascular lesion progression (Damiani et al., 2025). However, evidence linking TBPH exposure to atherosclerosis remains limited.

This study investigated the relationship between TBPH exposure and high-fat diet–induced atherosclerosis by integrating network toxicology, machine learning, molecular docking, and molecular dynamics (MD) simulations. Network toxicology translates multi-toxicant, multi-target interactions into visual networks and establishes a “toxicant–key gene–disease” framework, enabling in-depth analysis of protein–protein interactions (PPI) and pollutant-induced perturbations in molecular signaling pathways (Xie et al., 2025). Molecular docking predicts the interactions between small molecules and target proteins to estimate binding affinity (Chen et al., 2023), whereas MD simulations capture molecular behavior over time to evaluate the dynamic stability of molecular complexes (Meng et al., 2026). Multidimensional network analysis has attracted increasing attention because it integrates traditional toxicology with modern bioinformatics, thereby strengthening the validation of network toxicology findings. This approach can elucidate complex biomolecular networks, identify toxicity-related targets, and predict underlying molecular mechanisms, providing valuable insights into the health risks associated with contemporary environmental pollution (Xu et al., 2025).

The present study identified potential targets associated with TBPH and high-fat diet–induced atherosclerosis using network toxicology and machine learning. Molecular docking and MD simulations were also used to characterize the interactions between TBPH and candidate target proteins. Our findings are expected to explore the association between TBPH exposure and high-fat diet–induced atherosclerotic lesions.

## 2 Materials and methods

### 2.1 Toxicological analysis of TBPH

The three-dimensional chemical structure and canonical SMILES sequence of TBPH were obtained from the PubChem database. The toxicity profile of TBPH was predicted using ADMETlab 3.0 and ProTox-II.

### 2.2 TBPH target identification

Potential TBPH targets were predicted using the SwissTargetPrediction, TargetNet, and SEA databases, with the species restricted to *Homo sapiens*. Specifically, SwissTargetPrediction was used to identify TBPH targets with a probability score ≥0. TargetNet was used to screen targets with a prediction score ≥0.5, and SEA was used to identify target proteins with restricted to *Homo sapiens*. After integrating the results from the three databases, duplicate entries were removed, and the remaining targets were standardized using the IDMapping service. The final list of potential TBPH targets was obtained for subsequent intersection analysis.

### 2.3 Atherosclerosis target identification

Atherosclerosis-related targets were compiled by querying the GeneCards and OMIM databases using the keyword “atherosclerosis.” A relevance score cutoff of ≥3 was applied in GeneCards to prioritize candidates associated with atherosclerosis for analyses. Subsequently, UniProt standardization was performed for gene identifiers, resulting in a curated reference library of atherosclerosis-associated genes.

### 2.4 Identification of overlapping genes between TBPH targets and atherosclerosis targets

The intersection of TBPH- and atherosclerosis-associated targets was identified, and the overlapping genes were defined as potential targets associated with both TBPH exposure and atherosclerosis.

### 2.5 Establishment of the protein–protein interaction network

The 108 overlapping genes associated with both TBPH exposure and atherosclerosis were submitted to STRING database to evaluate PPI using a medium confidence threshold (0.4), restricted to *H. sapiens*, with all other parameters retained at their default settings. This threshold was selected to construct a comprehensive yet functionally relevant PPI network linking atherosclerotic pathogenesis to TBPH toxicity. Cytoscape (v3.9.1) was used to visualize the resulting PPI network. These 108 overlapping genes were expected to exhibit atherosclerosis-related functional interactions, thereby justifying the construction of a PPI network for identifying key hub regulators. Subsequently, the top 20 genes with the highest Matthews correlation coefficient (MCC) scores were identified based on network topological features using the CytoHubba plugin and were defined as hub genes for further analysis. The MCC algorithm was selected for hub gene identification because of its superior reliability and robustness, with all parameters maintained at their default settings.

### 2.6 Functional annotation and pathway enrichment analysis

To characterize the signaling pathways underlying TBPH-associated atherosclerosis, Gene Ontology (GO) and Kyoto Encyclopedia of Genes and Genomes (KEGG) pathway enrichment analyses were performed using Metascape. For each enriched GO term and KEGG pathway, several key enrichment parameters were computed, including the adjusted *P*-value (corrected for multiple testing using the Benjamini–Hochberg method) and gene ratio, among others. All significantly enriched GO terms and KEGG pathways were visualized using R software (v4.4.1).

### 2.7 Identification of key genes by intersecting differentially expressed genes with overlapping TBPH and atherosclerosis-associated genes

The GSE43292 dataset, consisting of 32 atherosclerotic lesions and matched adjacent non-lesioned vascular tissues from the same patients, was downloaded from the Gene Expression Omnibus database. This dataset has been extensively used for identifying differentially expressed genes (DEGs) between atherosclerotic and normal tissues (Gao et al., 2025; Zhang W. L. et al., 2025; Liu et al., 2026; Yan et al., 2026; Yu et al., 2026). Raw data were downloaded, processed, and normalized using the GEOquery and limma packages in R (v4.4.1). Data quality was evaluated by assessing normalization efficiency and sample distribution, and differential expression analysis was performed using the limma package in R, with thresholds set at |log fold change (FC)| ≥ 1 and adjusted *P*-value <0.05. All differential expression analyses were performed using paired statistical models in limma to account for the matched sample design. Volcano plots and heatmaps were generated using the ggplot2 and pheatmap packages in R. The resulting DEGs were consistent with those reported in previous studies, supporting the reliability of this present reanalysis. Subsequently, key target genes were identified by intersecting the genes associated with TBPH exposure and atherosclerosis (Section 2.4) with the DEGs identified from the GSE43292 dataset.

### 2.8 Machine learning and receiver operating characteristic (ROC) analysis

To screen candidate genes potentially associated with atherosclerosis, random forest (RF) and support vector machine–recursive feature elimination (SVM-RFE) algorithms were applied using the R packages randomForest [v4.7.1.1] and e1071 [v1.7.13], respectively (Sanz et al., 2018; Zhang J. Q. et al., 2025). Only transcripts meeting the differential expression criteria were retained as input features to reduce dimensionality and overfitting during machine learning. A fixed random seed of 42 was used throughout the analyses to ensure reproducibility. For RF modeling, the hyperparameters were set as follows: ntree = 100, mtry equal to the square root of the total number of input features, and min. node.size = 1. Model convergence and stability was assessed by monitoring the out-of-bag error and class-specific classification errors as the number of trees increased from 1 to 100. Feature importance was quantified using the MeanDecreaseAccuracy and MeanDecreaseGini indices. For SVM-RFE, the radial basis function kernel was adopted. Grid search combined with fivefold cross-validation was conducted to optimize the hyperparameters, yielding an optimal cost = 16 and gamma = 0.0625. Single-feature iterative elimination was performed, recording fivefold cross-validation classification error after each round to determine the optimal gene subset. The lowest cross-validation error was 0.27. Repeated stratified fivefold cross-validation was used for internal validation for both algorithms while preserving balanced case–control ratios to avoid sampling bias. The feature screening workflow was repeated 10 times with the same hyperparameters to evaluate stability, including classification consistency across replicates and gene importance ranking robustness. The frequency of each transcript among top-ranked feature sets was recorded across all runs. Multiple complementary metrics from the confusion matrix were calculated for comprehensive evaluation. Genes that consistently ranked highly in RF and SVM-RFE analyses in repeated validations were identified. ROC analysis was performed using the ROCR package, with the area under the curve (AUC) used as the performance metric.

### 2.9 Molecular docking analysis

The crystal structures of proteins encoded by core DEGs were retrieved from the Protein Data Bank (PDB). The three-dimensional structure of TBPH was downloaded from PubChem and subjected to energy minimization using the MMFF94 force field. All molecular docking calculations were performed using AutoDock Vina (v1.2.3). Before docking, protein structures were pretreated using PyMOL (v2.5.2), including the removal of water molecules, redundant salt ions, and endogenous miscellaneous small molecules to eliminate nonspecific binding interference. A global grid box enclosing the entire protein structure was constructed to perform full-surface blind docking. The preprocessed receptor and ligand structures were converted to AutoDock Vina-mandatory PDBQT format using ADFRsuite (v1.0.3). During docking, the global search exhaustiveness was set to 100, while all other parameters were maintained at their default values. The docking conformation with the highest docking score was selected as the optimal binding conformation for subsequent analysis. Finally, PyMOL was used to visualize the protein–ligand binding modes and interacting residues.

### 2.10 MD simulations

To evaluate the conformational stability and dynamic binding of the MMP9-TBPH complex, MD simulations were performed using AMBER 24. The optimal protein–ligand complex obtained from molecular docking was used as the initial simulation structure. Prior to simulation, the restrained electrostatic potential (AM1-BCC) charges of TBPH were calculated using the antechamber module. The ff14SB and GAFF2 force fields were applied to describe the MMP9 protein and the TBPH molecule, respectively. All hydrogen atoms were added using the LEaP module. A truncated octahedral TIP3P solvent box was constructed with a buffer distance of 10 Å between the complex and the box boundary to provide a sufficient solvent environment. Na^+^ and Cl^−^ ions were added for system charge neutrality, and the necessary topological and parameter files required for MD simulations were generated. The whole system underwent energy minimization to resolve atomic steric conflicts, starting with 2,500 steps of steepest descent followed by 2,500 steps of conjugate gradient. After energy minimization, a 200-ps heating simulation was performed under constant volume conditions to gradually increase the temperature from 0 to 298.15 K. Subsequently, a 500-ps NVT ensemble simulation was conducted at 298.15 K to achieve uniform solvent distribution, followed by a 500-ps NPT ensemble equilibrium simulation to stabilize system pressure and density. Finally, a 100-ns production MD simulation was performed under periodic boundary conditions in the NPT ensemble. During the simulation, the nonbonded cutoff distance was set to 10 Å. The Particle Mesh Ewald method was used to compute long-range electrostatic interactions, and the SHAKE algorithm was applied to constrain hydrogen bond lengths. The Langevin algorithm with a collision frequency of 2 ps^−1^, and the system pressure was maintained at 1 atm. A simulation time step was set to 2 fs, and trajectory files were saved every 10 ps for subsequent structural and energetic analysis. Root-mean-square deviation (RMSD), root-mean-square fluctuation (RMSF), radius of gyration (Rg), solvent-accessible surface area (SASA), hydrogen bond occupancy, and free-energy landscape (FEL) analyses were calculated to assess the structural stability and dynamic binding of protein–ligand complex.

### 2.11 MM/GBSA binding free energy calculation

The binding free energy between the protein and ligand was estimated using the molecular mechanics/generalized Born surface area (MM/GBSA) method. MD trajectories of 90–100 ns were used for this calculation. The corresponding computational formulas are as follows:

$$\Delta G_{\text{bind}}=\Delta G_{\text{complex}}-\left(\Delta G_{\text{receptor}}+\Delta G_{\text{ligand}}\right)=\Delta E_{\text{internal}}+\Delta E_{\text{VDW}}+\Delta E_{\text{elec}}+\Delta G_{\text{GB}}+\Delta G_{\text{SA}}.$$

### 2.12 Screening of key gene-related chemicals and drugs

Protein–chemical and protein–drug associations were identified using the NetworkAnalyst platform (https://www.networkanalyst.ca/). Drugs associated with the core genes were examined using the DSigDB database. Currently, DSigDB hosts 22,527 gene sets, including 17,389 drugs and 19,531 genes. An adjusted *P*-value <0.05 was used as the statistical criterion for identifying drugs associated with the target genes.

### 2.13 Chemicals and reagents

TBPH was purchased from Aladdin Industrial Corporation (Shanghai, China) with purity >98%. Dimethyl sulfoxide (DMSO) served as the vehicle for TBPH at a final concentration of 0.5% (v/v).

### 2.14 Establishment of atherosclerosis model and examination of TBPH effects

ApoE^−/−^ mice (male, 8 weeks; 22–25 g), a well-characterized model for atherosclerosis studies (Wen et al., 2024; Yuan et al., 2026), were obtained from Cyagen Biosciences Inc (Shanghai, China) and housed under a 12-h/12-h light–dark cycle in a temperature-controlled animal facility. All experimental procedures involving animals were approved by the Shanghai Tongren Hospital. The work has been reported in accordance with the ARRIVE guidelines (Animals in Research: Reporting *In Vivo* Experiments). Mice were randomly assigned to three treatment groups using random number table after 1 week of adaptive feeding (control atherosclerosis group, low-dose TBPH, and high-dose TBPH; n = 8 mice per group). All mice were fed a high-fat diet for 4 months to induce atherosclerosis, and the indicated treatment was co-administered once weekly by oral gavage. The control atherosclerosis group received vehicle, the low-dose TBPH exposure group received 20 mg/kg body weight [b.w.] TBPH, and the high-dose TBPH exposure group received 200 mg/kg b. w. TBPH. These exposure doses were adopted based on previous findings (Yin and Zhang, 2021). Briefly, human serum TBPH concentrations of approximately 260 ng/g lipid weight (He et al., 2013). With a 25% whole-body fat assumption, this biomonitoring value translates to a human equivalent oral dose of 1 mg/kg b. w. Based on the validated human-mouse allometric conversion factor of 12.3 (Reagan-Shaw et al., 2008), the baseline equivalent dose for mice was 12.3 mg/kg b. w. To account for uncertainties in extrapolation parameters and variable human exposure levels, 20 mg/kg b. w. was selected as toxicological experimental dose. By contrast, the 200 mg/kg b. w. high dose was used to explore the molecular and pathological mechanisms of TBPH. This high dose does not reflect real human exposure but amplifies biological changes to facilitate mechanistic analysis, as reported in earlier rodent studies of TBPH (Zhou et al., 2025). Blood samples were collected following terminal anesthesia, and aortic tissues were isolated for further analyses.

### 2.15 Histopathology and immunohistochemical staining

All quantitative analyses were performed on whole aortic tissues. Paraffin-embedded sections (7 μm) were prepared from mouse tissues using standard procedures and subjected to hematoxylin and eosin (H&E) staining to assess aortic morphology and the atherosclerotic lesion ratio was calculated as lesion area divided by the total vessel wall area. Masson’s trichrome staining to quantify collagen deposition, and the collagen area fraction was determined by normalizing collagen-positive area to the total vessel area. The frozen sections (7 μm) were prepared for Oil Red O staining to evaluate lipid accumulation, and the lipid deposition ratio was obtained by calculating the lipid-positive area relative to the total aortic area. For immunohistochemical (IHC) analysis, the sections were incubated overnight at 4 °C with a primary antibody against MMP9, followed by incubation with a horseradish peroxidase (HRP)-conjugated secondary antibody for 2 h at room temperature. Subsequently, the sections were developed with 3,3′-diaminobenzidine (Beyotime Biotechnology, China) and counterstained with hematoxylin. Images of the stained sections were captured using a confocal laser scanning microscope (Leica DMi8, Leica Microsystems, Wetzlar, Germany) equipped with a bright-field transmission channel for imaging all staining, including DAB immunohistochemical stains. Five serial sections from each mouse were stained and quantitatively analyzed.

### 2.16 Measurement of serum lipids and inflammatory cytokines

Serum total cholesterol (TC), triglyceride (TG), and low-density lipoprotein cholesterol (LDL-C) levels were measured using commercial kits following the indicated treatment protocols (Nanjing Jiancheng Bioengineering Institute, Nanjing, China). Serum concentrations of matrix metalloproteinase 9 (MMP9), tumor necrosis factor-α (TNF-α), and interleukin-6 (IL-6) were quantified using enzyme-linked immunosorbent assay kits using a microplate reader (Bio-Rad, Hercules, CA, United States).

### 2.17 Statistical analysis

All experimental results were analyzed using GraphPad Prism. Normally distributed continuous variables are presented as mean ± standard deviation. Shapiro–Wilk normality test and Levene’s test for equal variance were performed to validate the prerequisites of statistical tests before intergroup comparison. Student’s t-test was used for comparisons between two groups, while one-way analysis of variance followed by Tukey’s *post hoc* test was applied for comparisons across multiple groups. Tukey’s multiple comparison correction was implemented when analyzing multiple parallel biological endpoints. Statistical significance was defined as *P* < 0.05.

## 3 Results

### 3.1 Toxicological analysis of TBPH

The toxicity profile of TBPH was evaluated using the ADMETlab 3.0 and ProTox-3.0 databases (Table 1). Both platforms indicated that TBPH has nephrotoxic potential. Furthermore, TBPH was associated with a substantial risk of hepatotoxicity, neurotoxicity, and carcinogenicity. Overall toxicity was classified as Grade 5, and a human oral median lethal dose of 5,000 mg/kg was predicted using the ProTox-II-3.0 platform. These findings highlight the potential health risks of TBPH and provide a basis for further investigation into its mechanisms of toxicity.

**TABLE 1:** Results of TBPH toxicity.

Database results		
Property	ProTox-3.0 prediction	ADMETlab3.0 toxicity probability
Carcinogenicity	-	0.335
Hepatotoxicity	Inactive	0.054
Neurotoxicity	Inactive	0.331
Nephrotoxicity	Active	0.052
Respiratory toxicity	Inactive	-
Cardiotoxicity	Inactive	-

### 3.2 Potential targets of TBPH-associated atherosclerosis

**FIGURE 1:**
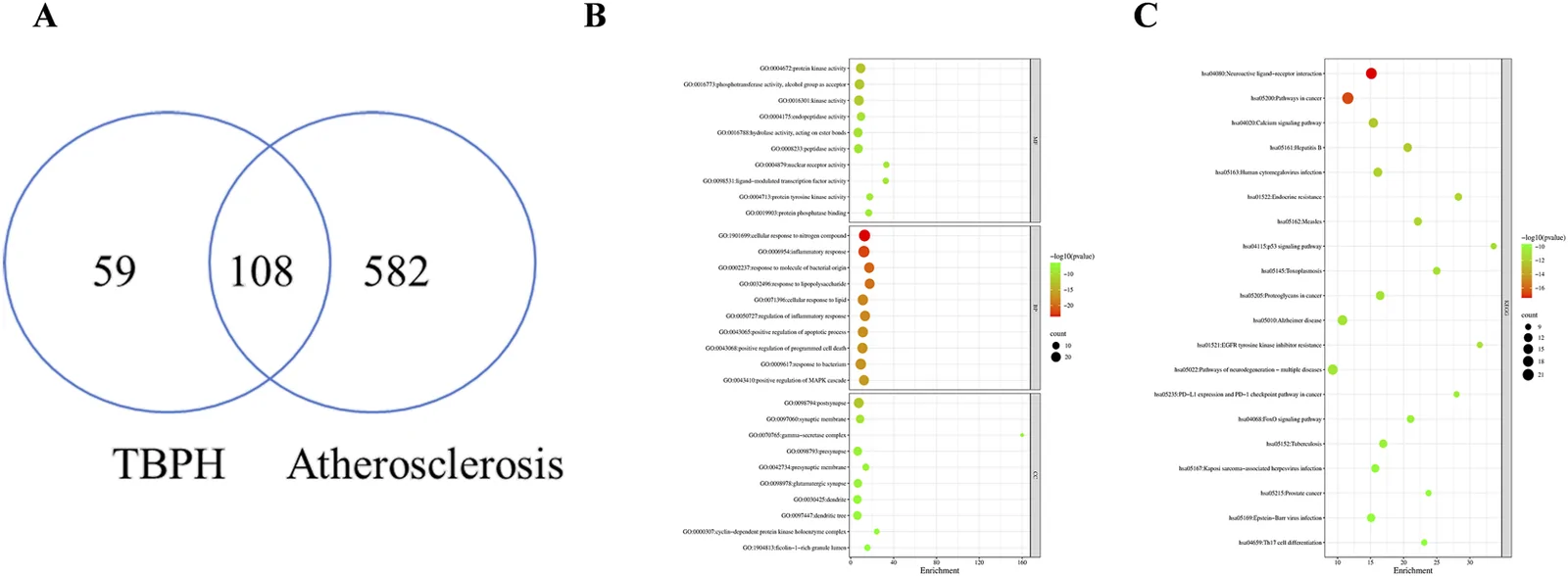
Identification of TBPH target genes and the key roles in atherosclerosis. **(A)** Venn diagram showing overlapping genes between TBPH-related targets and atherosclerosis-associated genes, which were defined as the targets of TBPH in atherosclerosis. **(B,C)** Functional enrichment analyses of the overlapping targets, with bubble plots displaying the significantly enriched Gene Ontology (GO) terms and Kyoto Encyclopedia of Genes and Genomes (KEGG) pathways.

To identify candidate targets linking TBPH exposure and atherosclerosis, 167 putative TBPH-related targets were predicted using SwissTargetPrediction, TargetNet, and SEA databases. Concurrently, 690 atherosclerosis-related targets were retrieved from the GeneCards and OMIM databases. Venn diagram intersection analysis identified 108 overlapping genes, which were used as candidate targets related to TBPH toxicity and atherosclerotic pathogenesis (Figure 1A).

To characterize the biological functions and signaling pathways associated with these 108 overlapping candidate genes, GO analysis revealed enrichment in protein kinase activity, inflammatory response, response to lipopolysaccharide, cellular response to lipids, positive regulation of the apoptotic process, postsynaptic components and other biological processes and molecular functions (Figure 1B; Supplementary Figure S1A). These findings suggest the potential involvement of the identified genes in immune activation and apoptotic signaling pathways. Moreover, KEGG analysis indicated that the 108 overlapping targets were enriched in apoptosis- and antioxidant stress–related pathways, particularly the p53 (p.adjust = 2.61443 × 10^-5^) and FoxO signaling (p.adjust = 9.57213 × 10^−5^; Figure 1C; Supplementary Figure S1B). These results suggest that TBPH exposure may be associated with cellular apoptosis during atherosclerosis. Notably, enriched signaling pathways show only transcriptomic correlations with TBPH treatment; their functional regulatory effects require experimental verification.

**FIGURE 2:**
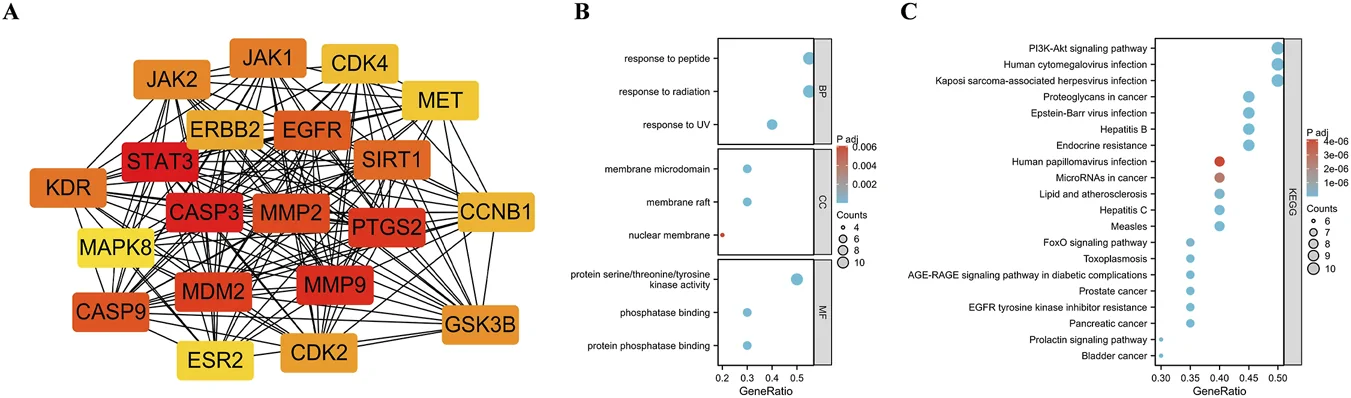
Construction of the protein–protein interaction (PPI) network and identification of hub genes. **(A)** Top 20 hub genes screened from the PPI network using the CytoHubba plugin based on MCC scores. **(B,C)** Bubble plots illustrating the significantly enriched GO terms and KEGG pathways for the top 20 hub genes.

To explore the functional relationships among the 108 overlapping targets, a PPI network was constructed using STRING, comprising 108 nodes and 197 edges with an average node degree of 3.65 (Supplementary Figure S2). To further identify the hub genes from the PPI network, the top 20 hub genes were identified, including caspase-3 (*CASP3*), epidermal growth factor receptor (*EGFR*), prostaglandin-endoperoxide synthase 2 (*PTGS2*), matrix metalloproteinase 9 (*MMP9*), signal transducer and activator of transcription 3 (*STAT3*), mitogen-activated protein kinase 8 (*MAPK8*), glycogen synthase kinase 3 beta (*GSK3B)*, sirtuin 1 (*SIRT1*), erb-b2 receptor tyrosine kinase 2 (*ERBB2*), MDM2 proto-oncogene (*MDM2*), caspase 9 (*CASP9*), matrix metallopeptidase 2 (*MMP2*), mitogen-activated protein kinase 14 (*MAPK14*), kinase insert domain receptor, cyclin-dependent kinase 4 (*CDK4*), cyclin-dependent kinase 2 (*CDK2*), Janus kinase 2 (*JAK2*), protein tyrosine phosphatase receptor type C (*PTPRC*), recombinant human estrogen receptor beta (*ESR2*), and Janus kinase 1 (*JAK1*) (Figure 2A).

Subsequent GO enrichment analysis revealed that these 20 hub genes were primarily associated with peptide responses, membrane raft localization, and serine/threonine/tyrosine kinase activity (Figure 2B). KEGG pathway analysis highlighted enrichment in the PI3K-Akt signaling pathway (p.adjust = 2.91336 × 10^−8^), lipid and atherosclerosis-associated pathways (p.adjust = 1.70075 × 10^−7^), and FoxO pathways (p.adjust = 1.40348 × 10^−7^, Figure 2C), supporting potential correlations among TBPH and apoptosis-associated processes during atherosclerosis.

### 3.3 Intersection analysis of DEGs and overlapping genes associated with TBPH exposure and atherosclerosis and validation of key gene expression

**FIGURE 3:**
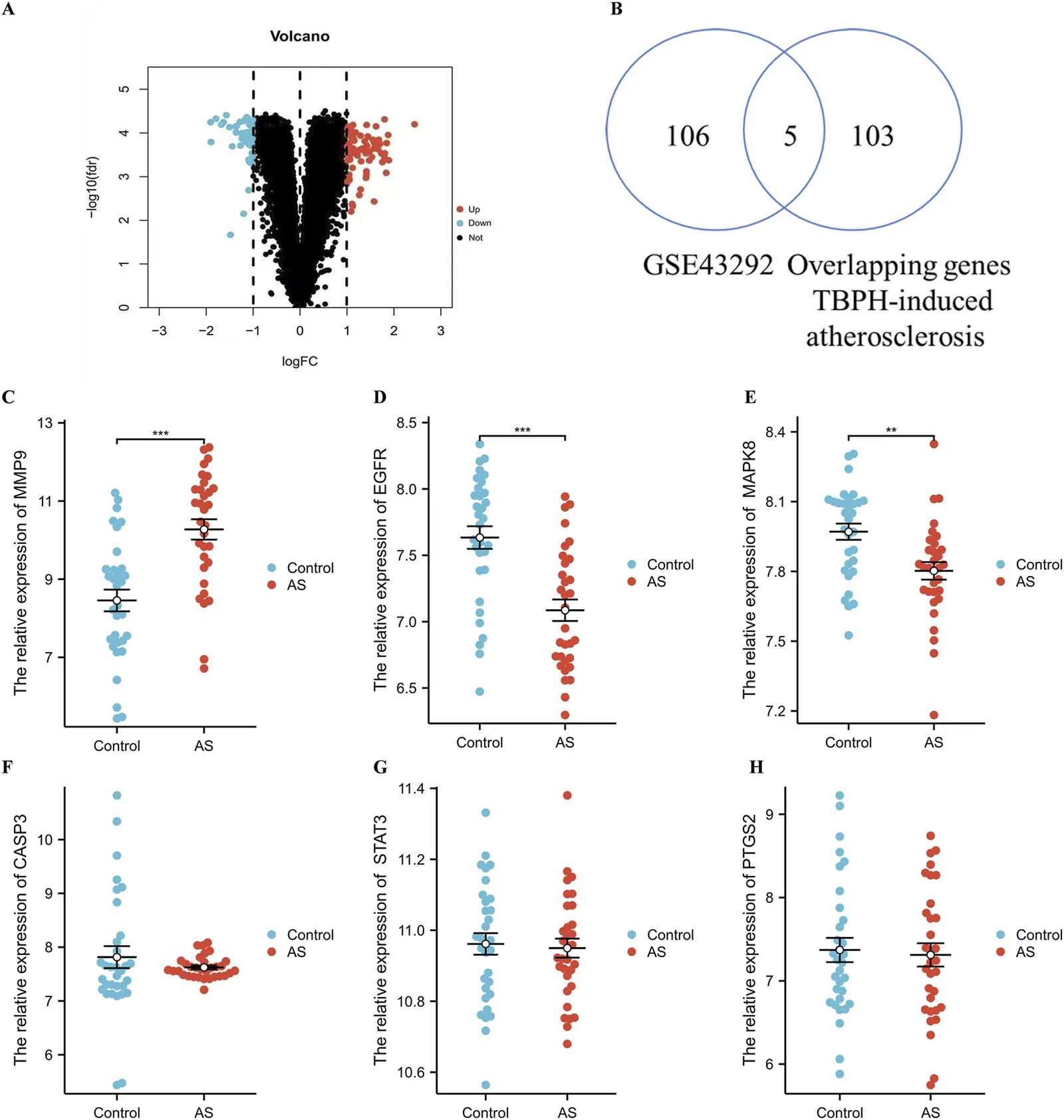
Intersection analysis of DEGs and overlapping genes and validation of key gene expression. **(A)** Volcano plot showing differentially expressed genes (DEGs) between the healthy and atherosclerosis groups from the GSE43292 dataset. **(B)** Venn diagram displaying common targets between DEGs and the overlapping genes of TBPH-related targets and atherosclerosis-associated genes. **(C–H)** Transcriptional profiles of the key candidate genes were analyzed from the public GSE43292 dataset.

Differential expression analysis of the GSE43292 dataset yielded 111 DEGs (56 upregulated and 55 downregulated), which were visualized via a volcano plot (Figure 3A; Supplementary Figure S3). Candidate target genes were defined as the intersection between the 108 overlapping genes implicated in both TBPH exposure and atherosclerosis with the 111 DEGs, including *MMP9*, *EGFR*, *ANPEP*, *THRB*, and *MAPK8* (Figure 3B). Notably, *MMP9*, *EGFR*, and *MAPK8* were among the top 20 hub genes (Figure 2A).

Expression validation in GSE43292 demonstrated that *MMP9* was markedly upregulated (Figure 3C), whereas *EGFR* and *MAPK8* were downregulated (Figures 3D,E) in atherosclerotic tissues compared with those in healthy controls. In contrast, *CASP3*, *PTGS2*, and *STAT3* showed no significant changes (Figures 3F–H). These findings highlight *MMP9*, *EGFR*, and *MAPK8* as candidate mediators that may contribute to the atherogenic effects associated with TBPH exposure.

**FIGURE 4:**
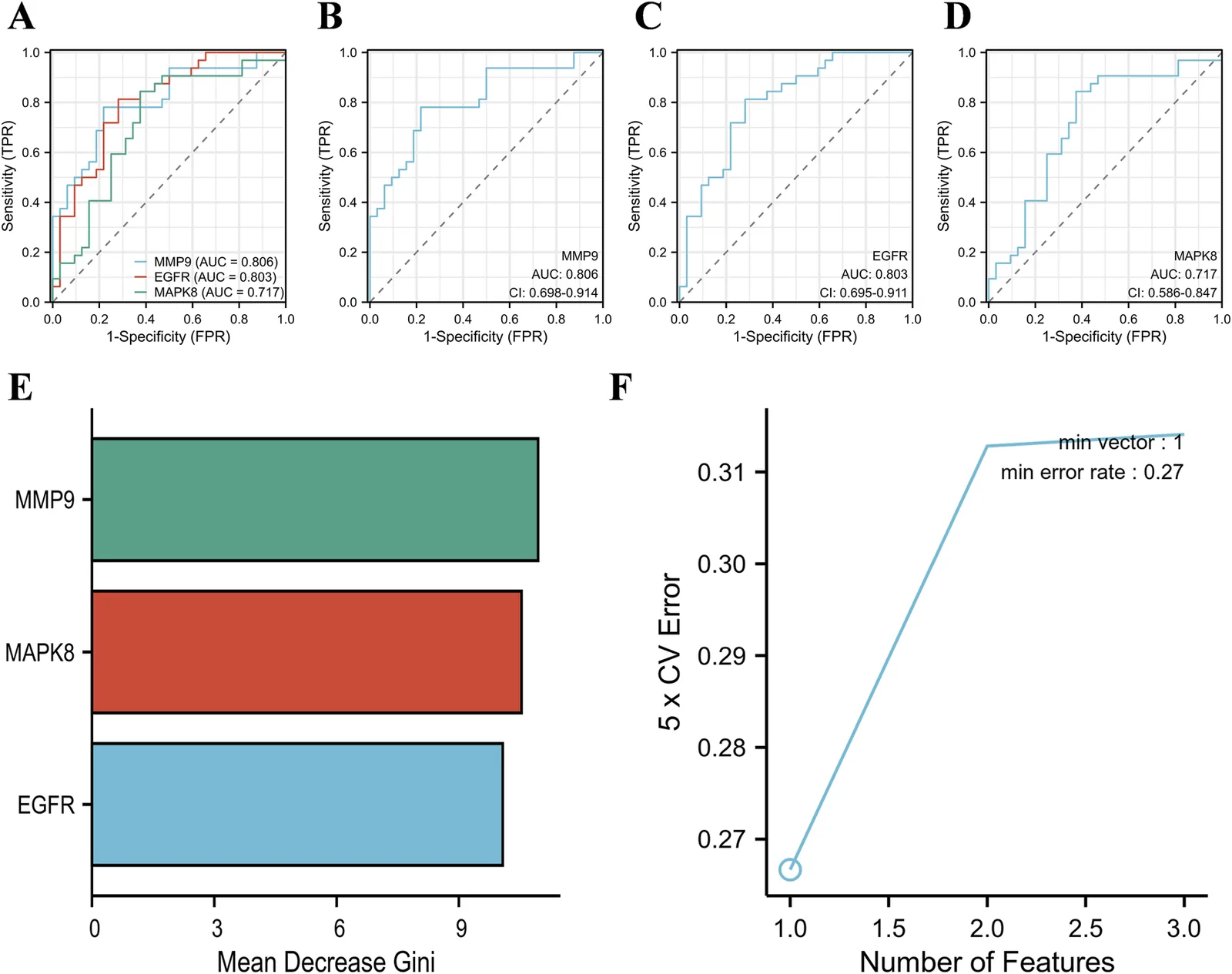
Identification of the key candidate gene. **(A)** Receiver operating characteristic (ROC) curve analysis of the key genes. **(B–D)** ROC curves of MMP9, EGFR, and MAPK8, respectively. **(E)** Feature importance ranking of atherosclerosis-related indicators prioritized MMP9 using the random forest (RF) method. **(F)** Validation of the key genes performed by the support vector machine-recursive feature elimination (SVM-RFE) method.

Moreover, ROC curve analysis showed AUC values of 0.806 for *MMP9*, 0.803 for *EGFR*, and 0.717 for *MAPK8* (Figures 4A–D), which only indicates predictive ability within this cohort. The machine learning framework served only as an exploratory tool to prioritize potential targets. RF analysis presented *MMP9* among the highest-ranked candidate features (Figure 4E), and SVM-RFE screening similarly prioritized *MMP9* at the top of the gene list (Figure 4F). Integrated bioinformatic data indicates *MMP9* represents a candidate molecule correlated with atherosclerotic lesions.

### 3.4 Molecular docking of candidate proteins and TBPH

**FIGURE 5:**
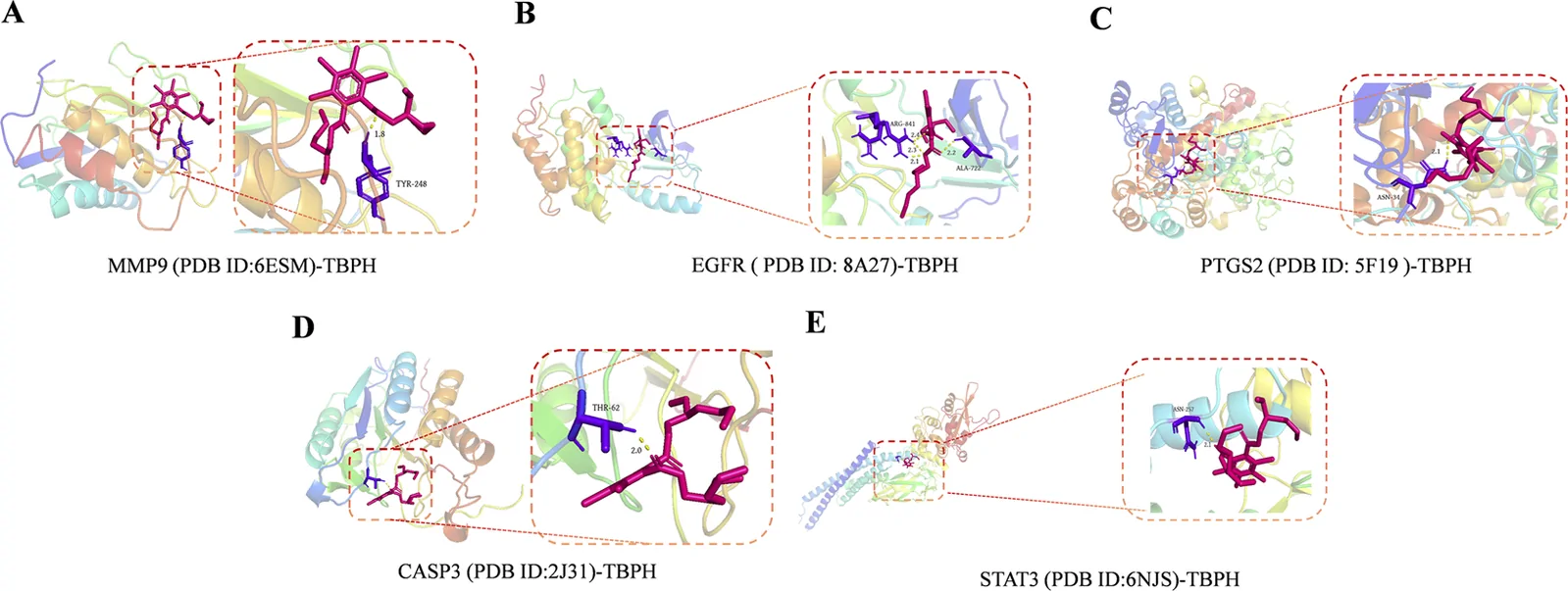
Molecular docking analysis of the lowest binding energy interactions between TBPH and key target proteins. **(A–E)** Predicted binding affinities of TBPH with MMP9 **(A)**, EGFR **(B)**, PTGS2 **(C)**, CASP3 **(D)**, and STAT3 **(E)**, respectively.

Molecular docking was performed to explore the interactions between TBPH and candidate proteins. The results showed the following binding energies: MMP9, −7.61 kcal/mol (PDB ID: 6ESM); EGFR, −5.87 kcal/mol (PDB ID: 8A27); PTGS2, −5.52 kcal/mol (PDB ID: 5F19); CASP3, −4.8 kcal/mol (PDB ID: 2J31), and STAT3, −4.73 kcal/mol (PDB ID: 6NJS). A binding energy threshold of −5.0 kcal/mol was used as a preliminary screening criterion to screen targets with relatively high predicted binding affinity, consistent with widely used standards in previous study (Trott and Olson, 2010; Li Y. W. et al., 2025). Among these complexes, the TBPH–MMP9 complex showed the lowest docking score, suggesting stronger predicted binding affinity. The lowest-energy docking conformations for all TBPH–protein complexes (MMP9, EGFR, PTGS2, CASP3, and STAT3) were visualized using PyMOL (Figures 5A–E; Supplementary Figure S4A). Furthermore, TBPH bound to the active site of the MMP9 protein, with its stable complex mainly maintained through hydrophobic interactions. It formed hydrophobic contacts with multiple key residues of MMP9, including VAL-101, PHE-107, LEU-267, LEU-234, HIS-230, PHE-110, and PRO-193. Among these, the aromatic residues (PHE-107 and PHE-110) and hydrophobic residues (VAL and LEU) created a hydrophobic binding microenvironment (Supplementary Figure S4A). Collectively, the *in silico* analyses indicate binding affinity between TBPH and the candidate target proteins, with MMP9 exhibiting the lowest binding free energy.

### 3.5 MD simulations of the interaction between MMP9 and TBPH

**FIGURE 6:**
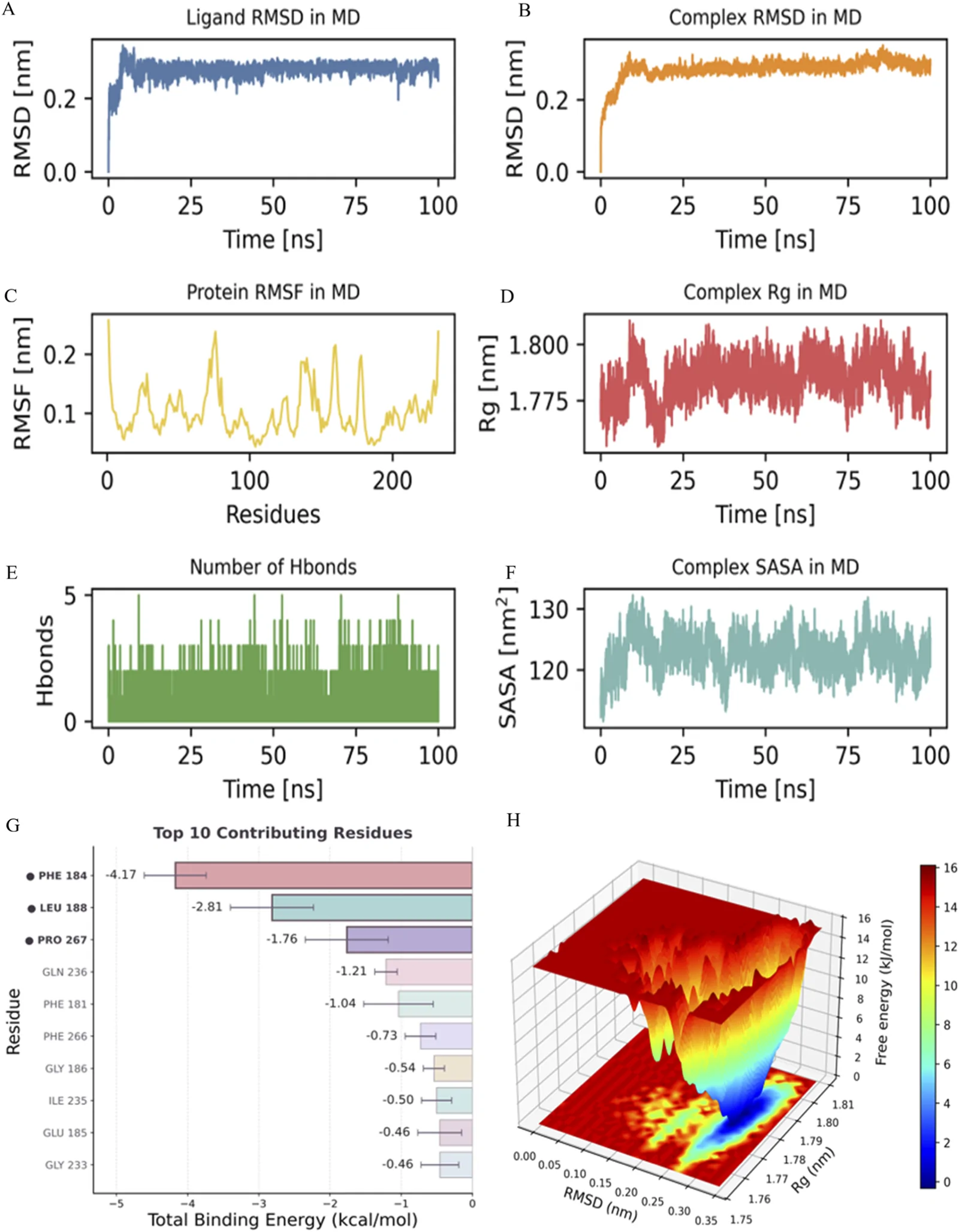
Molecular dynamics (MD) simulation of the TBPH-MMP9 complex to evaluate structural stability and interaction strength. **(A)** Ligand RMSD; **(B)** Whole complex RMSD; **(C)** Residue-wise RMSF of MMP9; **(D)** Radius of gyration (Rg) of the complex; **(E)** Time series of hydrogen bond counts between protein and ligand; **(F)** Solvent-accessible surface area (SASA) of the complex; **(G)** MM/GBSA residue energy decomposition showing the top 10 key stabilizing residues (binding energy in kcal/mol); **(H)** Free-energy landscape (FEL) plotted against complex RMSD and Rg, highlighting the dominant low-energy stable conformation of the complex.

MD simulation analyses, including ligand RMSD, complex RMSD, protein RMSF, Rg, hydrogen bond count, SASA, and FEL, were performed to evaluate the dynamic stability and conformational properties of the MMP9–TBPH complex (Figure 6). Ligand RMSD increased rapidly at the initial simulation stage and stabilized after approximately 10 ns, fluctuating within 0.25–0.30 nm throughout (Figure 6A), indicating that TBPH adjusted its conformation and maintained a stable binding pose. The complex RMSD also reached equilibrium after 10 ns and remained stable at 0.25–0.30 nm (Figure 6B), demonstrating system stability. RMSF results showed that most protein residues had low flexibility between 0.05–0.15 nm (Figure 6C), indicating that TBPH binding stabilized MMP9. Complex Rg values stayed within 1.76–1.80 nm with minor fluctuations (Figure 6D), showing that the system remained compact and well-folded throughout the simulation. Hydrogen bond count fluctuated between 1 and 5 (Figure 6E), with reversible hydrogen bond formation and breakage indicating polar interactions between TBPH and the MMP9 binding pocket. SASA stabilized at 120–130 nm^2^ after initial structural adaptation (Figure 6F), reflecting a stable state.

Based on MD trajectories, the MM/GBSA method was employed to calculate binding free energy, with the binding energy components and overall binding energy of the MMP9–TBPH complex summarized in Table 2. The complex’s total binding free energy (ΔG) was −41.07 ± 2.99 kcal/mol, showing potential binding affinity between TBPH and MMP9. Van der Waals energy (ΔE = −49.84 ± 2.364 kcal/mol) served as the predominant driving force for complex binding, whereas electrostatic, polar solvation, and nonpolar solvation energies collectively modulated binding behavior. Residue-level analysis of the key amino acids affecting ligand binding (Figure 6G) showed that PHE184 contributed most to stability, followed by LEU188 and PRO267, with their negative free energies enhancing TBPH binding stability through van der Waals interactions, hydrophobic effects, and local spatial matching. The residues GLN236, PHE181, PHE266, GLY186, ILE235, GLU185, and GLY233 provided moderate stabilization, maintaining a stable microenvironment within the binding pocket. Aromatic residues (e.g., PHE184, PHE181, and PHE266) strengthened ligand localization via hydrophobic stacking and aromatic ring interactions, whereas hydrophobic residues (e.g., LEU188 and ILE235) stabilized ligand embedding by providing a nonpolar binding microenvironment. The combined effects of key residues ensured stable binding in the MMP9–TBPH complex. FEL analysis revealed a distinct low-free-energy basin centered on the complex, forming a deep, continuous energy valley (Figure 6H). Combined with the RMSD and Rg results, the FEL distribution confirmed MMP9–TBPH complex structural stability throughout the simulation. Collectively, these results highlight the MMP9–TBPH complex’s stability and dynamic behavior under physiological simulation conditions.

**TABLE 2:** Binding free energies and energy components predicted by MM/GBSA (kcal/mol).

System name	MMP9/TBPH
ΔE_vdw_	−49.84 ± 2.364
ΔE_elec_	−4.87 ± 1.12
ΔG_GB_	19.22 ± 1.19
ΔG_SA_	−5.58 ± 0.33
ΔG_bind_	−41.07 ± 2.99

ΔE_vdW_: van der Waals energy.

ΔE_elec_: electrostatic energy.

ΔG_GB_: electrostatic contribution to solvation.

ΔG_SA_: non-polar contribution to solvation.

ΔG_bind_: binding free energy.

### 3.6 Prediction of drugs targeting key genes

Protein–chemical interaction networks were constructed via NetworkAnalyst based on the CTD database to generate exploratory hypotheses. The raw network contained 897 nodes and 2,218 edges (Supplementary Figure S5A), and a simplified subnetwork with 23 nodes and 84 edges was retained (Supplementary Figure S5B). In addition, NetworkAnalyst combined with the DrugBank database was applied to build protein–drug interaction networks. The original network included 145 nodes and 140 edges (Supplementary Figure S5C), which was further trimmed into a concise subnetwork containing 11 nodes and 8 edges (Supplementary Figure S5D). DSigDB was used to compound computationally predicted to interact with each hub gene, yielding 25 entries associated with MMP9, 187 with EGFR, 45 with CASP3, 117 with PTGS2, and 30 with STAT3. Among these predicted compounds, marimastat was retrieved from both databases as a molecule with predicted binding capacity toward MMP9. However, it should be emphasized that marimastat functions as a broad-spectrum pan-MMP inhibitor rather than a MMP9-specific agent.

### 3.7 TBPH correlates with aggravated atherosclerotic lesions under high-fat diet conditions

**FIGURE 7:**
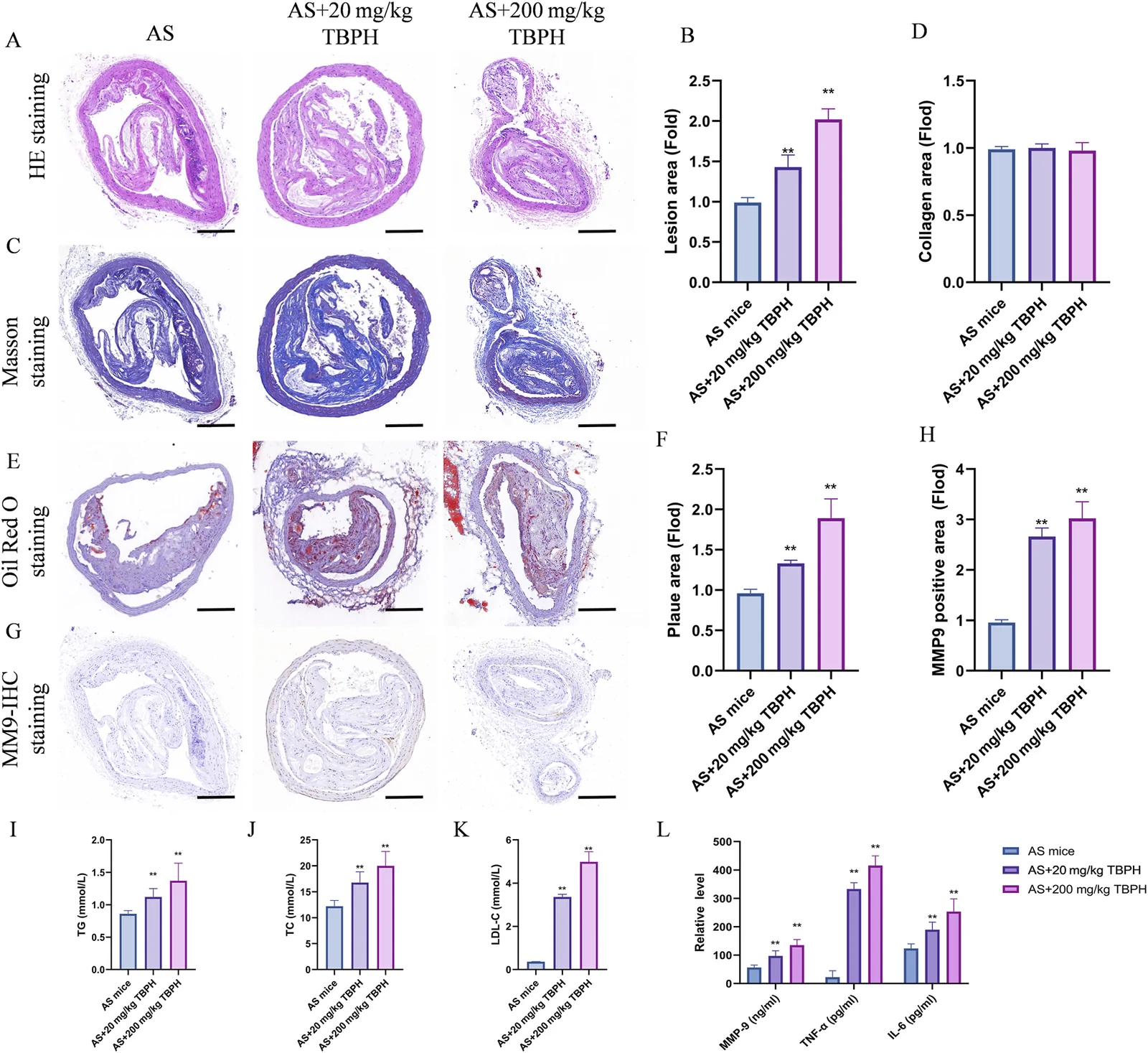
Correlation of TBPH exposure with atherosclerotic progression and MMP9 expression under high-fat diet conditions. **(A,B)** Representative hematoxylin-eosin (H&E) staining images of whole aortic sections (n = 8 mice per group); scale bar = 100 μm. **(C,D)** Representative Masson’s trichrome staining images of whole aortic sections (n = 8 mice per group); scale bar = 100 μm. **(E,F)** Representative Oil Red O staining images of whole aortic sections (n = 8 mice per group); scale bar = 100 μm. **(G,H)** Immunohistochemical (IHC) staining analysis illustrating MMP9 expression in whole aortic sections (n = 8 mice per group); scale bar = 100 μm. **(I–K)** Serum levels of total cholesterol (TC), triglycerides (TG), and low-density lipoprotein cholesterol (LDL-C) in mice. **(L)** Serum levels of interleukin-6 (IL-6), tumor necrosis factor-α (TNF-α), and matrix metalloproteinase-9 (MMP9) in mice (n = 8 mice per group), measured via enzyme-linked immunosorbent assay (ELISA). Data are presented as mean ± SD of three independent experiments. P < 0.01 vs. the control group (ApoE^−/−^ mice fed a high-fat diet).

To further elucidate the potential association between TBPH exposure and atherosclerosis under high-fat diet conditions, vascular tissues stained with H&E, Masson’s trichrome, and Oil Red O were analyzed to compare vascular lesions among the control, low-dose TBPH, and high-dose TBPH groups. Under high-fat diet conditions, both dose TBPH exposure may aggravate atherosclerotic lesion formation (Figures 7A–F). Consistent with *in silico* predictions, IHC analysis revealed upregulation of MMP9 expression in aortic tissues (Figures 7G,H), where the high-dose TBPH group exhibited stronger MMP9 expression. These data suggest a correlative association between TBPH exposure, increased MMP9, and atherosclerotic lesion formation under high-fat diet conditions.

To examine the effects of TBPH exposure on lipid metabolism, a key determinant of atherosclerosis risk, serum TC, TG, and LDL-C levels were compared across experimental groups. Compared with the control group, both dose TBPH exposure raised serum TC, TG and LDL-C levels, with greater lipid accumulation observed in the high-dose group (Figures 7I–K). Furthermore, the serum concentrations of MMP9 and proinflammatory cytokines IL-6 and TNF-α were also increased, with the high-dose TBPH group demonstrating the higher elevations (Figure 7L). Collectively, these *in vivo* observations reveal that TBPH exposure correlates with aggravated atherosclerotic lesions and increased MMP9 levels under high-fat diet conditions.

## 4 Discussion

The widespread manufacture, ubiquitous environmental distribution, and persistence TBPH have raised growing toxicological concerns. Human exposure to TBPH primarily occurs via inhalation, oral ingestion, and dermal absorption (Hoffman et al., 2014; La Guardia et al., 2017). Existing preclinical evidence has documented the multi-organ toxicity of TBPH. It disrupts thyroid and gonadal function in rodents (Springer et al., 2012); promotes hepatic lipid accumulation and nonalcoholic fatty liver disease (NAFLD)-like phenotypes through PPARγ- or FOXO1-dependent signaling pathways in zebrafish (Zhou et al., 2024); and worsens hepatic steatosis and inflammation in mouse models of nonalcoholic steatohepatitis (Guo et al., 2021). Prior *in vitro* studies have also shown that TBPH is associated with injury to human vascular endothelial cells (Xiang et al., 2017); however, research on the molecular mechanisms linking TBPH exposure to atherosclerosis remains limited. This study applied an integrated method combining network toxicology, transcriptomic validation, molecular docking, and MD simulations was used to investigate the potential association between TBPH exposure and atherosclerosis.

Potential molecular targets of TBPH were predicted using SwissTargetPrediction, TargetNet, and SEA databases, whereas atherosclerosis-related targets were retrieved from the GeneCards and OMIM databases, yielding 108 overlapping genes associated with TBPH toxicity and atherosclerosis. Topological analysis of the PPI network constructed from these 108 overlapping genes identified 20 hub genes, and subsequent machine learning analyses prioritized MMP9 as a candidate target for further analyses. Molecular docking and MD simulations predicted that TBPH forms favorable binding interactions with MMP9. MMP9 drives vascular injury by mediating extracellular matrix breakdown. For instance, MMP9 contributes to atherosclerosis in the Ldlr^−/−^Apob100/100 mouse model (Wågsäter et al., 2011). Activated MMP9 can degrade collagen and other extracellular matrix components, leading to structural changes in the arterial wall (Sabry et al., 2021; Theofilis et al., 2022). Elevated MMP9 levels have also been associated with plaque instability, highlighting its critical role in atherogenesis (Zhang et al., 2021; Zivkovic et al., 2024). Overall, these findings indicate a correlation between elevated TBPH exposure, atherosclerotic lesions and increased MMP9 levels under high-fat diet conditions. Nevertheless, the current study did not determine whether MMP9 is a causal factor in the development of atherosclerotic lesions or merely serves as an inflammatory biomarker. Additional histological staining is needed to better understand the microstructure of these lesions and locate MMP9 within cells, which will help clarify its regulatory role.

Several limitations of the study should be acknowledged. First, we only identified correlations among TBPH exposure, atherosclerotic lesions, and MMP9 upregulation without establishing causality. The high-fat diet–induced atherosclerotic model differs pathologically from the direct atherogenic effects of TBPH. Additionally, our MMP9 screening results lack validation from external transcriptomic data and clinical cohorts. Cellular experiments are needed to confirm direct interactions and functional regulation. Moreover, unquantified serum and tissue TBPH concentrations limit the study’s translational relevance to human exposure. In summary, our findings indicate a potential association between TBPH and high-fat diet–related atherosclerosis, although further functional studies are required to confirm its causal role.

## 5 Conclusion

This study provides multidimensional analysis indicating that TBPH may be associated with atherosclerosis progression, in conjunction with increased MMP9 expression under high-fat diet conditions. Our findings refine the current understanding of the toxicological profile of TBPH and provide valuable evidence for environmental risk assessment, while also establishing a foundation for future mechanistic research.

## Data Availability

The original contributions presented in the study are included in the article/Supplementary Material, further inquiries can be directed to the corresponding author.
